# Assessment of functionality and scientific evidence of mobile health applications (mHealth apps) for people with dementia and their caregivers

**DOI:** 10.1192/j.eurpsy.2022.450

**Published:** 2022-09-01

**Authors:** M. Zeiler, C. Chmelirsch, P.L. Kolominsky-Rabas

**Affiliations:** 1 Department of Medical Informatics, Friedrich-alexander-university Erlangen-nürnberg, Erlangen, Germany; 2 Interdisciplinary Center for Health Technology Assessment (HTA) and Public Health (IZPH), Friedrich-alexander-university Erlangen-nürnberg, Erlangen, Germany; 3 Digital Dementia Registry Bavaria (digiDEM Bayern), Interdisciplinary Center for Health Technology Assessment (HTA) and Public Health (IZPH), Friedrich-alexander-university Erlangen-nürnberg, Erlangen, Germany

## Abstract

**Introduction:**

There is a rapid increase in the use of mHealth apps to provide care and support to people with deminetia (pwd) and their caregivers.

**Objectives:**

Functionality as well as scientific evidence of mHealth apps were analysed from a Health Technology Assessment (HTA) perspective

**Methods:**

mHealth apps for pwd and their caregivers were identified in the app stores and assessed for functionality and methodological quality of the underlaying scientific evidence. Functionality was assessed with the *Mobile App Rating Scale-German (MARS- G*), methodological quality of studies using the *Critical Appraisal Skills Program (CASP)* checklists.

**Results:**

A total of 20 mHealth apps for were identified via systematic search in the *Apple App Store* and the *Google Play Store.* The overall quality of the apps can be rated medium with a mean score of 3.38. Studies have been published for only 30% of the apps (n = 6). 13 studies were included in the assessment of methodological quality, but the app itself was the object of study in only 2 publications. In summary methodological weaknesses such as small group sizes, a short study duration and insufficient comparative therapy were frequent.

**Conclusions:**

Our results demonstrate that the majority of apps is lacking reliable scientific evidence. To support pwd and caregivers as end users in their choice standardised HTA criteria has to be defined and applied for mHealth apps.

The *Digital Dementia Registry Bavaria (digiDEM Bayern*) is funded by the Bavarian Ministry of Health (No.: G42d-G8300-2017/1606-381).

PROSPERO Registration Number: CRD42021260309.

**Disclosure:**

No significant relationships.

